# microRNA-9 might be a novel protective factor for osteoarthritis patients

**DOI:** 10.1186/s41065-020-00128-y

**Published:** 2020-04-22

**Authors:** Lei Jiang, Xu Sun, Hongyang Kong

**Affiliations:** Department of Orthopedics, Taizhou People’s Hospital, No. 366 Taihu Road, Taizhou City, 225300 Jiangsu Province China

**Keywords:** Osteoarthritis, microRNAs, Protein-protein interaction, Modules, protective factor

## Abstract

**Background:**

The study aimed to identify the targeting genes and miRNAs using the microarray expression profile dataset for Osteoarthritis (OA) patients. Differentially expressed genes (DEGs) between OA and control samples were identified using Bayes method of limma package. Subsequently, a protein-protein interaction (PPI) network was constructed. miRNAs and transcription factor (TFs) based on DEGs in PPI network were identified using Webgestalt and ENCODE, respectively. Finally, MCODE, Gene Ontology (GO) function, and Kyoto Encyclopedia of Genes and Genomes (KEGG) were performed. The expressions of several DEGs and predicted miRNAs in OA rats were detected by RT-PCR.

**Results:**

A total of 594 DEGs were identified. In PPI network, there were 313 upregulated DEGs and 22 downregulated DEGs. Besides, the regulatory relationships included 467 upregulated interactions and 85 downregulated interactions (miR-124A → *QKI* and *MAP 1B*) between miRNA and DEGs in PPI network. The module from downregulated DEGs-TFs-miRNA networks was mainly enriched to low-density lipoprotein particle clearance, response to linoleic acid, and small molecule metabolic process BP terms. Moreover, *QKI*, *MAP 1B* mRNA and miR-9 expressions were significantly reduced in OA rats.

**Conclusion:**

miR-9 might be a protective factor for OA patients via inhibiting proliferation and differentiation of cartilage progenitor cells. miR-124A might play an important role in progression of OA through targeting *QKI* and *MAP 1B*.

## Background

Osteoarthritis (OA) is the most prevalent rheumatic disease with the characteristics of chronic, debilitating and degenerative disease of the joints [1]. Approximately 1/3 of OA patients is over 60 years old [2]. Besides, OA often occurs in people aged 60–70 years, more frequently in females (18%) than males (10%) [1] . Currently, treatments are only used to manage symptoms due to complex etiology [3]. Although some pathogenesis pathways have been reported, current study is incomplete for prevention or treatment. These limitations might be surmounted through investigating the molecular mechanisms in OA.

microRNAs (miRNAs), as novel regulators of gene expression, play key roles in biological processes of OA [4]. Previous study has found that some miRNAs associates with musculoskeletal system [5–7]. miR-140 is involved in the cartilage of mouse embryos during the development of long and flat bone through directly targeting distone deacetylase 4 (*HDAC4*) [8]. In addition, miR-140 protects against OA development by targeting ADAM metallopeptidase with thrombospondin type 1 motif 5 (*Adamts-5*) expression [9]. The tumor necrosis factor a (TNF-α) is an important inflammatory factor, and its expression level is increased in OA synovial membranes [10]. Decreased expression of miR-130a, correlated with TNF-α in the development of OA [6]. Thus, miR-140 and miR-130a are novel therapeutic targets in OA development. Therefore, it is necessary to identify more miRNAs associated with OA development in the future.

The present study aimed to identify the targeting genes and miRNAs using the microarray expression profile dataset. The differentially expressed genes (DEGs) were identified using Bayes method of limma package. Subsequently, a protein-protein interaction (PPI) network for these DEGs was constructed. miRNAs and transcription factor (TFs) based on DEGs in PPI network were identified using Webgestalt and ENCODE, respectively. Finally, significant network modules were obtained by MCODE, and their Gene Ontology (GO) function and Kyoto Encyclopedia of Genes and Genomes (KEGG) were enriched.

## Results

### The results of DEGs involved in the function and pathway for OA

Totally, 594 DEGs were identified between OA and control samples, including 535 upregulated DEGs (*MMP2, FOS,* et al) and 59 downregulated DEGs (*QKI, MAPB1,* et al). The up-regulated DEGs were enriched to 1166 BP terms, 20 KEGG pathways, and 83 Reactome pathways. The top 5 of these were showed in Table 1. The down-regulated DEGs were enriched to 480 BP terms, 4 KEGG pathways, and 27 Reactome pathways. The top 5 of these were showed in Table 2.

**Table 1:** The top 5 of biological processes (BP) terms, Kyoto Encyclopedia of Genes and Genomes (KEGG) pathways, and Reactome pathways for upregulated differentially expressed genes (DEGs)

**GO_ID**	**BP term Pathway**
GO:0030198	extracellular matrix organization
GO:0009888	tissue development
GO:0045597	positive regulation of cell differentiation,
GO:0007155	cell adhesion
GO:0022610	biological adhesion
**KEGG_ID**	**KEGG Pathway**
04350	TGF-beta signaling pathway
04510	Focal adhesion
04142	Lysosome
04512	ECM-receptor interaction
05110	Vibrio cholerae infection
**Reactome_ID**	**Reactome Pathway**
1,442,490	Collagen degradation
3,000,170	Syndecan interactions
216,083	Integrin cell surface interactions
1,474,228	Degradation of the extracellular matrix
1,474,244	Extracellular matrix organization

**Table 2:** The top 5 of BP terms, KEGG pathways, and Reactome pathways for downregulated DEGs

**GO_ID**	**BP term Pathway**
GO:0034439	lipoprotein lipid oxidation
GO:0006629	lipid metabolic process
GO:0044281	small molecule metabolic process
GO:0010876	lipid localization
GO:0044255	cellular lipid metabolic process
**KEGG_ID**	**KEGG Pathway**
00982	Drug metabolism - cytochrome P450
04975	Fat digestion and absorption
04920	Adipocytokine signaling pathway
03320	PPAR signaling pathway
**Reactome_ID**	**Reactome Pathway**
75,109	Triglyceride Biosynthesis
163,560	Hormone-sensitivelipase (HSL)-mediated triacylglycerol hydrolysis
535,734	Fatty acid, triacylglycerol, and ketone body metabolism
1,266,738	Developmental Biology,
381,340	Transcriptional regulation of white adipocyte differentiation

### The DEGs-TFs-miRNA integrated network

**Fig. 1:**
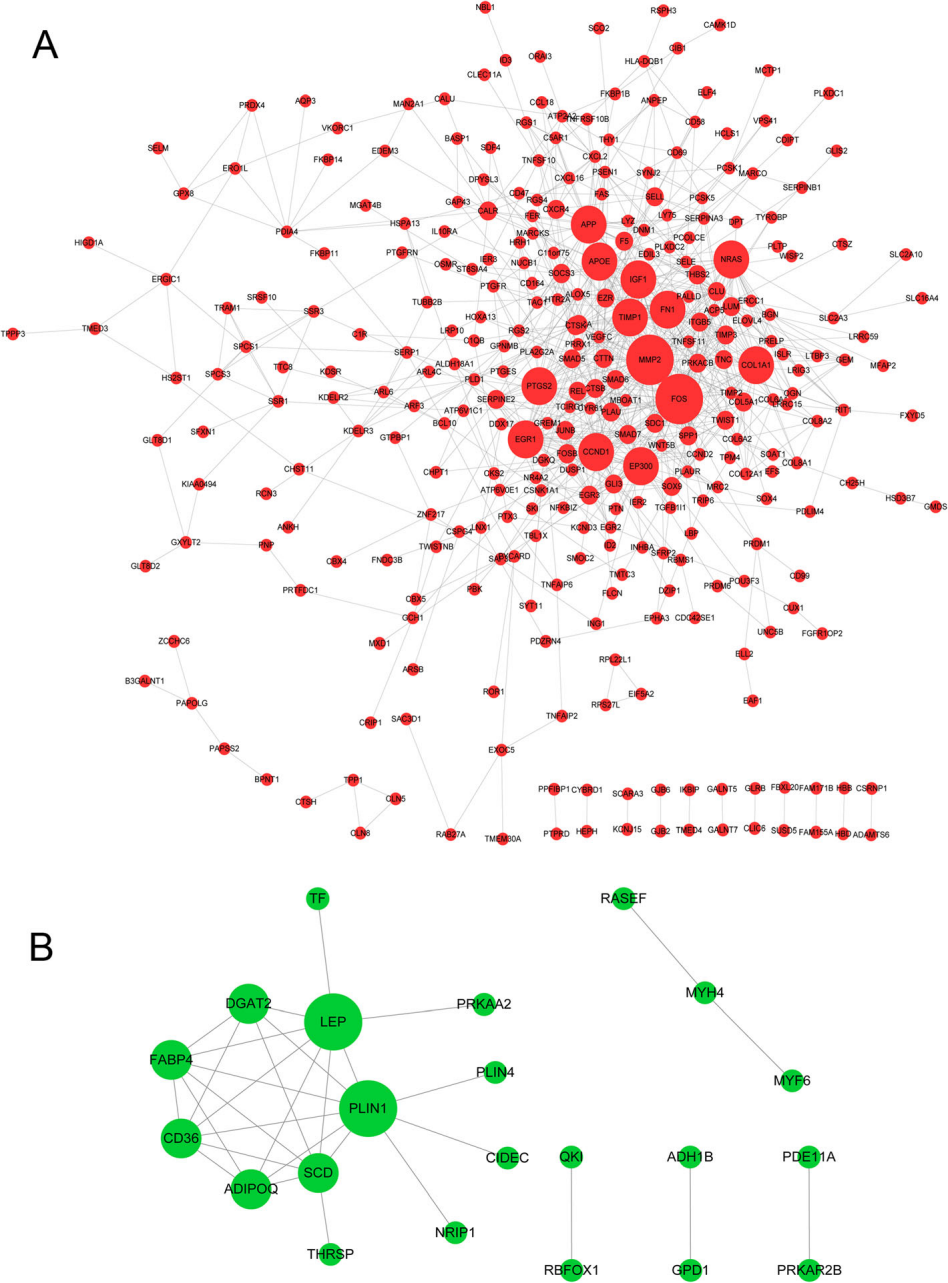
The protein-protein interaction (PPI) network for upregulated differentially expressed genes (DEGs) (**a**) and downregulated DEGs (**b**). Red nodes indicate upregulated DEGs, green nodes indicate downregulated DEGs, and the black lines indicate the interactions. The node is larger, and it degree is higher, suggesting that it has more interactions with other proteins in PPI network

**Fig. 2:**
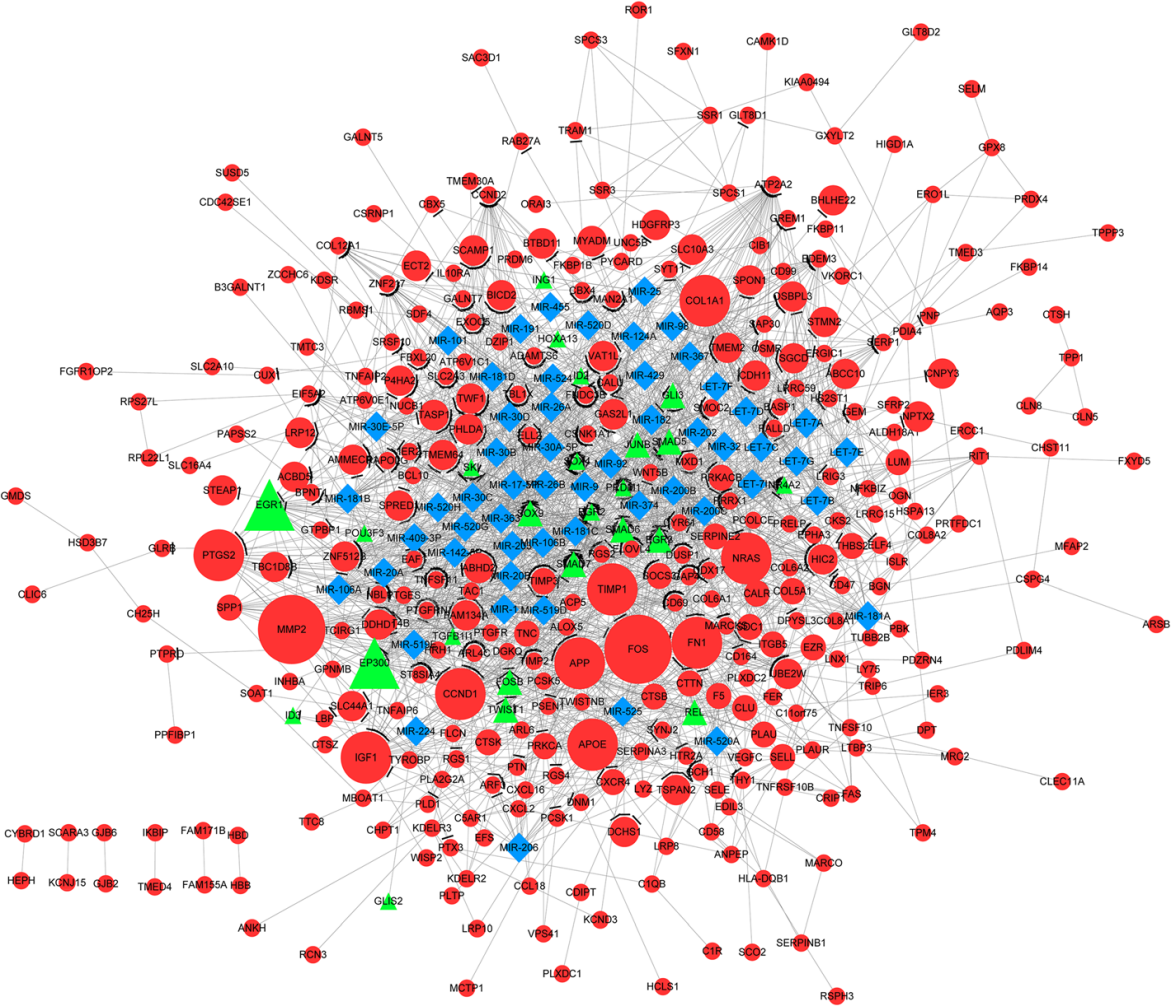
The integrated network based on upregulated DEGs, transcription factor (TFs), and miRNA. Red circles indicate DEGs, green triangles indicate TFs, and blue rhombus indicate miRNAs. Arrows indicate transcriptional regulatory relationship, T-type lines indicate miRNA regulation relationship, straight lines indicate PPIs

**Fig. 3:**
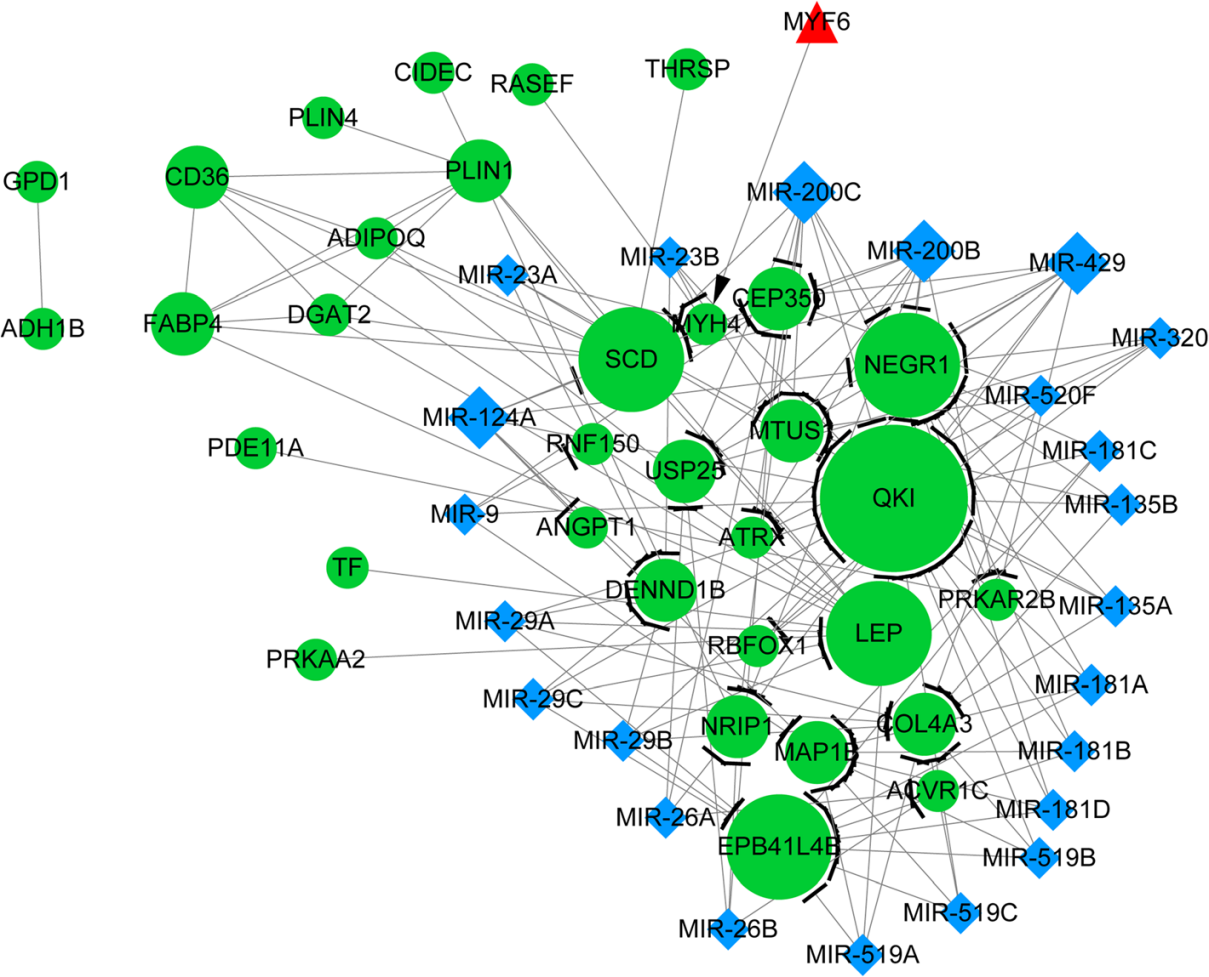
The integrated network based on downregulated DEGs, transcription factor (TFs), and miRNA. Green circles indicate DEGs, red triangles indicate TFs, and blue rhombus indicate miRNAs. Arrows indicate transcriptional regulatory relationship, T-type lines indicate miRNA regulation relationship, straight lines indicate PPIs

In PPI network, there were 313 upregulated DEGs with 841 interactions and 22 downregulated DEGs with 31 interactions (Fig. 1). Among them, 25 upregulated DEGs (*GLIS2, FOSB,* et al) and one downregulated DEG (*MYF6*) were also TFs, which have a transcriptional regulatory relationship with other DEGs. In addition, the regulatory relationships between miRNA and DEGs in PPI network were also identified (e.g. miR-124A → *QKI* and *MAP 1B*), including 467 upregulated interactions and 85 downregulated interactions. The DEGs-TFs-miRNA networks based on upregulated and downregulated DEGs are showed in Figs. 2 and 3, respectively. The nodes with degree more than 20 in upregulated DEGs-TFs-miRNA network are showed in Table 3, which presents 17 miRNAs (eg., miRNA-9) and 9 TFs. In addition, in the downregulated DEGs-TFs-miRNA network, *QKI* had the largest degree value (degree = 21) and only one MYF6 TF was predicted. There were 4 miRNAs (eg., miR-124A) and 11 DEGs (eg., *QKI and MAP 1B*) in top 15 nodes listed by degree (Table 4), and QKI and MAP 1B were both predicted as target of miR-124A.

**Table 3:** The nodes with degree more than 20 in the integrated network based on upregulated DEGs, transcription factor (TFs), and miRNA. The blod fonts indicate miRNA, the italic indicate TFs

Symbol	Degree	Symbol	Degree
FOS	51.0	**MIR-30D**	27.0
COL1A1	36.0	**MIR-30C**	27.0
CCND1	36.0	**MIR-30A-5P**	27.0
ATP2A2	35.0	*SMAD6*	26.0
APP	35.0	TIMP1	25.0
MMP2	34.0	PTGS2	25.0
NRAS	34.0	HIC2	25.0
FN1	34.0	FNDC3B	25.0
*SMAD7*	34.0	TIMP3	24.0
*EGR3*	33.0	PRRX1	24.0
**MIR-519D**	32.0	*SOX9*	23.0
**MIR-20B**	32.0	**MIR-524**	23.0
**MIR-106B**	32.0	**MIR-124A**	22.0
**MIR-106A**	32.0	*GLI3*	22.0
**MIR-20A**	32.0	**MIR-9**	22.0
**MIR-17-5P**	32.0	CCND2	22.0
*EGR1*	30.0	**MIR-429**	21.0
IGF1	29.0	**MIR-200C**	21.0
*NR4A2*	29.0	**MIR-200B**	21.0
*EP300*	28.0	PRKACB	21.0
APOE	27.0	EDEM3	21.0
**MIR-30E-5P**	27.0	*SOX4*	21.0
**MIR-30B**	27.0	ZNF512B	21.0

**Table 4:** The top 15 nodes ranked by degree values in the integrated network based on downregulated DEGs, transcription factor (TFs), and miRNA. The bold fonts indicate miRNA, the italic indicate TFs

Symbol	Degree
QKI	Degree: 21.0
SCD	Degree: 11.0
NEGR1	Degree: 11.0
LEP	Degree: 11.0
EPB41L4B	Degree: 10.0
**MIR-200B**	Degree: 9.0
**MIR-200C**	Degree: 9.0
**MIR-429**	Degree: 9.0
MAP 1B	Degree: 9.0
COL4A3	Degree: 9.0
PLIN1	Degree: 9.0
CEP350	Degree: 8.0
MTUS1	Degree: 8.0
**MIR-124A**	Degree: 7.0
USP25	Degree: 6.0

### The significant network modules in the integrated network

**Fig. 4:**
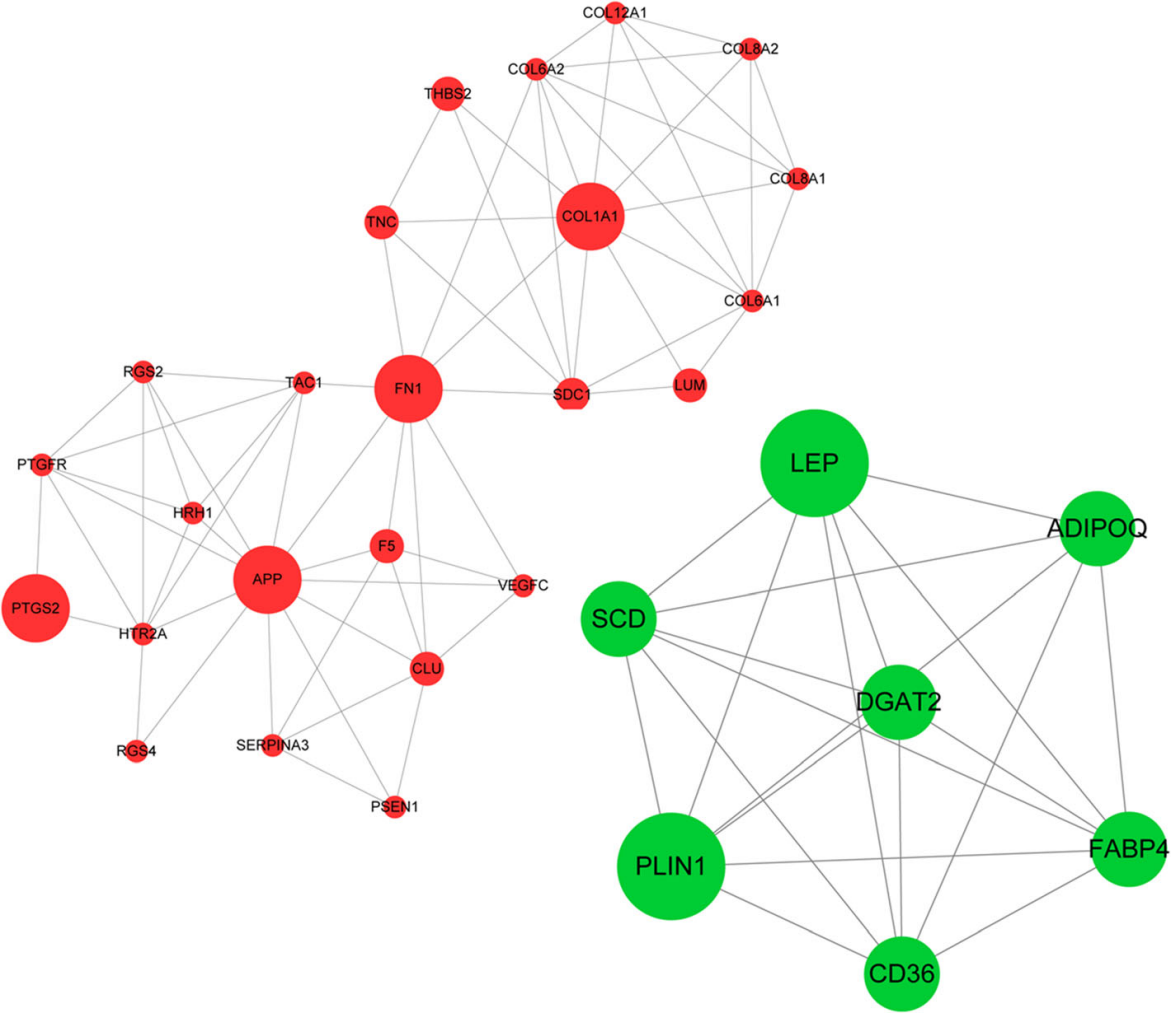
The significant network module with the highest Mcode score (5.739) from the upregulated DEGs-TFs-miRNA integrated network and the module (Mcode score = 6.667) from the downregulated DEGs-TFs-miRNA integrated network. Red nodes indicate upregulated DEGs, green nodes indicate downregulated DEGs

Overall, 12 modules were identified from the upregulated network. The module with the highest Mcode score (5.739) included 24 nodes and 66 interactions (Fig. 4a). These DEGs in this module were mainly enriched to extracellular matrix organization, extracellular matrix disassembly, and collagen catabolic process BP terms, as well as ECM-receptor interaction, focal adhesion, and PI3K-Akt signaling pathways. Moreover, only one module was obtained from the downregulated network (Mcode score = 6.667), including 7 nodes and 20 interactions (Fig. 4b). They were mainly enriched to low-density lipoprotein particle clearance, response to linoleic acid, and small molecule metabolic process BP terms.

### The results of qRT-PCR

**Fig. 5:**
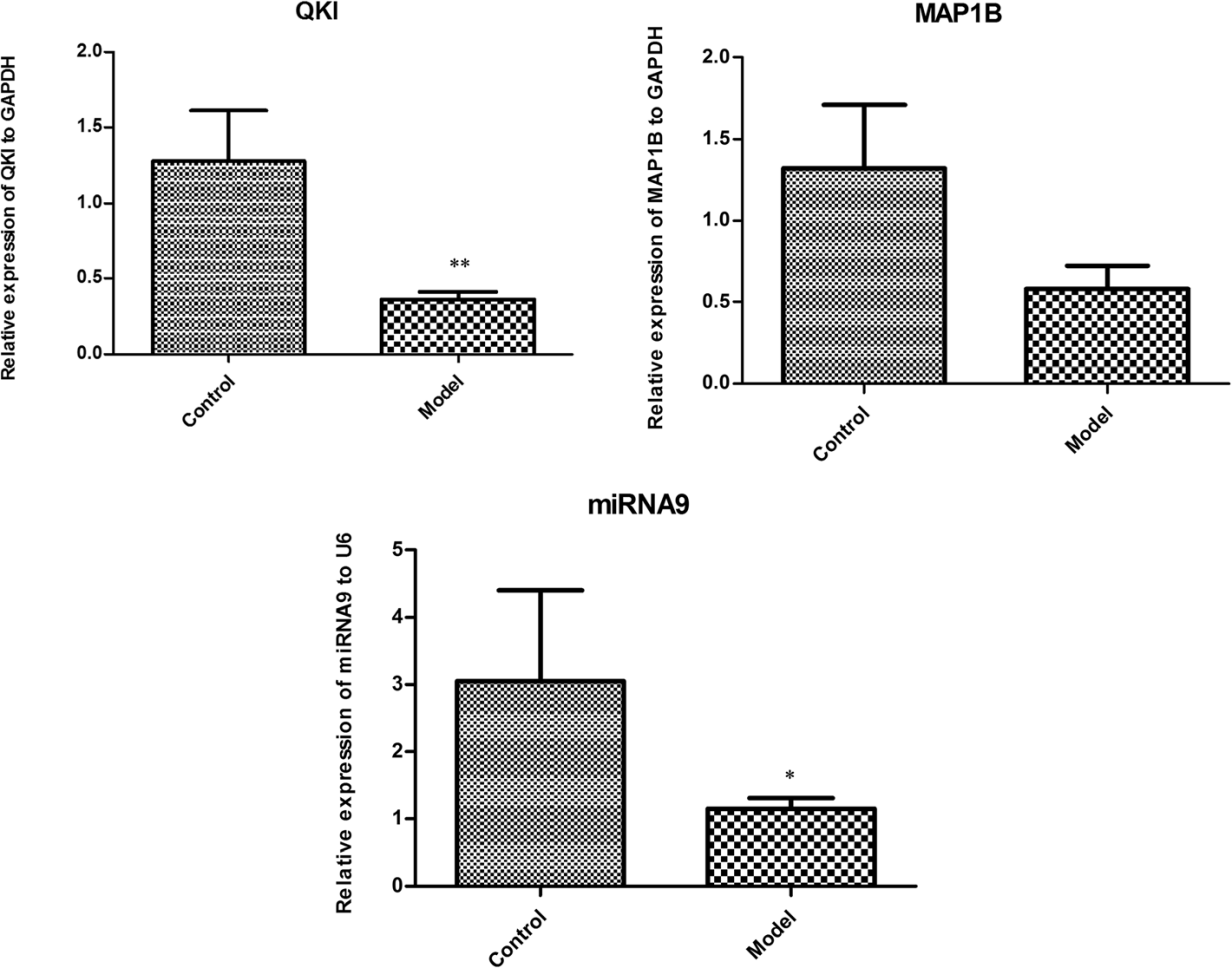
The relative expressions of miR-9, *QKI* and *MAP 1B*. *P < 0.05 indicates that there is significantly different between control and OA samples. **P < 0.01

As shown in Fig. 5, expressions of *QKI* Mrna (*P* < 0.01) and miR-9 (*P* < 0.05) were significantly reduced in OA rats. Although expression of *MAP 1B* was also reduced in synovial tissue of OA rats, there were no significant differences when compared with that of health rats.

## Discussion

In this study, 535 upregulated DEGs and 59 downregulated DEGs (e.g. *QKI* and *MAPB1*) were identified between OA and control samples. Among them, 313 upregulated DEGs with 841 interactions and 22 downregulated DEGs with 31 interactions were used to construct PPI network. As a result, 25 upregulated DEGs and one downregulated DEG in PPI network having a transcriptional regulatory relationship with other DEGs were obtained. In upregulated DEGs-TFs-miRNA network, miR-9 had higher degree values and was predicted to regulate 22 targets. Besides, the regulatory relationships (e.g. miR-124A → *QKI* and *MAP 1B*) were highlighted in downregulated DEGs-TFs-miRNA network, in which *QKI* had the largest degree value, and *QKI* and *MAP 1B* in top 15 nodes were both predicted as target of miR-124A. The module from downregulated DEGs-TFs-miRNA networks was mainly enriched to low-density lipoprotein particle clearance, response to linoleic acid, and small molecule metabolic process BP terms. Moreover, it was confirmed that expressions of *QKI, MAP 1B* mRNA and miR-9 were significantly reduced in OA rats.

As is described above, miRNAs are novel regulators of gene expression and play key roles in biological processes of OA. miR-9 expression is upregulated in osteosarcoma tissues when compared with human osteoblastic hFOB cells and the corresponding non-cancerous bone tissues [11]. Whereas osteosarcoma is derived from progenitor cells, which can be differentiated into bone, cartilage and fiber [12]. In other word, cartilage contain a number of stem cells or progenitor cells, which has been confirmed by a previous study [13]. In addition, Jiang et al. have found that cartilage-derived stem/progenitor cells might have a key role in cartilage repair, which will behave therapeutic potential in OA [14]. MiR-9 has been identified to play critical roles in proliferation and differentiation of retinal progenitor cell and neural progenitor cells [15, 16]. In a round-about way, miR-9 might play a consequence role in OA through regulating proliferation and differentiation of cartilage progenitor cell for cartilage repair. In the present study, miR-9 expression is reduced in OA rats when compared with the healthy rats, suggesting miR-9 is involved in pathological process of OA. Similarly, a previous study indicates that expression level of miR-9 is decreased in OA chondrocytes with numerous apoptotic cell deaths through upregulating PRTG [17]. Therefore, miR-9 might be a novel protective factor for OA patients via inhibiting proliferation and differentiation of cartilage progenitor cells.

In the present study, KH Domain Containing RNA Binding (QKI) and Microtubule Associated Protein 1B (MAP 1B) were predicted as target genes of miR-124A. miR-124 plays roles in various pathologic conditions such as inflammatory responses, and osteoclast differentiation [18] miR-124A is overexpressed in OA cartilage with cocultured chondrocytes derived from MSC [19, 20]. Notably, it shows the alterations in chondrogenesis and neurogenesis are associated with the development of OA [21]. QKI belongs to a family of RNA-binding proteins called STAR proteins for Signal Transduction and Activation of RNA [22]. Recently, a study has confirmed that *QKI* genes is significantly suppressed when synovial explants were treated with miR-27b-3p in OA patients [23]. *MAP 1B* belongs to the microtubule-associated protein family and is involved in microtubule assembly [24]. Actin cytoskeleton and microtubule array play essential roles on chondrogenesis [25]. It has been reported that expression of *MAP 1B* in mRNA and protein levels is significantly upregulated in degraded cartilage by Steinberg [26]. The 3′UTR of *MAP 1B* mRNA interacts with QKI resulted in reducing *MAP 1B* mRNA expression in the QKI-deficiency mutant mice. Meanwhile, RNAi-mediated QKI-knockdown caused destabilization of the *MAP 1B* mRNA in oligodendroglia CG4 cell line [27]. Furthermore, overexpression of exogenous *QKI* was able to facilitate *MAP 1B* expression. Interesting, *QKI* and *MAP 1B* mRNA expression levels were also reduced in rats with OA in this study. Therefore, we inferred miR-124A might play an important role in progression of OA through targeting *QKI* and *MAP 1B*.

However, there are still some limitations in the study. All of miRNAs in DEGs-TFs-miRNAs were predicted in OA samples using bioinformatic analysis, and their expressions and regulatory relationships should be verified using future experiments. In addition, these results will be confirmed using human samples. Moreover, the expression of *MAP 1B* was reduced in synovial tissue of OA rats than in health rats, but no significant differences, which should be verified in large samples in future.

## Conclusion

miR-9 might be a novel protective factor for OA patients OA synovia via inhibiting proliferation and differentiation of cartilage progenitor cells. In addition, miR-124A might play an important role in progression of OA through targeting *QKI* and *MAP 1B*. This study will provide important clues for understanding of the mechanisms of OA and the development of therapeutic targets for OA.

## Methods

### Data acquisition and preprocessing

The expression profile dataset GSE82107 was downloaded from the Gene Expression Omnibus (GEO; http://www.ncbi.nlm.nih.gov/geo/) database. The data was deposited into the following platform: GPL570 [HG-U133_Plus_2] (Affymetrix Human Genome U133 Plus 2.0 Array) by Marieke et al., including 10 end-stage OA synovial biopsies from the Orthopedics department of the Radboud University Medical Center and 7 synovial biopsies from individuals without a joint disease [28]. The study protocols were approved by CMO region Arnhem-Nijmegen in Netherlands.

### Identification of DEGs

The data was normalized using RMA function of Affy package in R software (http://www.bioconductor.org/packages/release/bioc/html/affy.html). Subsequently, DEGs between OA and control samples were identified using Bayes method of limma package in R software (http://www.bioconductor.org/packages/release/bioc/html/limma.html). The threshold value of DEGs was set as |log fold change (FC)| > 1.0 and *P* value < 0.05.

### GO enrichment function and pathway analyses

The GO function, KEGG pathway and Reactome pathway enrichment analyses were performed to reveal the DEGs involved in biological processes (BP) and pathways through an online database (www.biocloudservice.com). *P* value < 0.05 was used as the significant difference.

### Construction of the PPI network

Proteins rarely act alone as their functions tend to be regulated. However, PPI network can provide a valuable framework to understand the functional organization of the proteome [29]. Therefore, the PPIs of DEGs was analyzed using STRING online tool (version 10.0). DEGs with combined score > 0.4 were identified.

### Identification of miRNAs and TFs based on DEGs in PPI network

Webgestalt was used to identify miRNAs that regulated DEGs in PPI network under the threshold value of gene count ≥4 and adjusted *P* values < 0.05. In addition, TFs were screened from DEGs in the PPI network using ENCODE database [30].

### The integrated network using DEGs in the PPI network, miRNA, and TFs

DEGs in PPI network and regulation relationships of miRNA-DEGs and TFs-DEGs were integrated to a network using Cytoscape (version 3.2.0, http://www.cytoscape.org/). As we known, the integrated network subjected to scale-free network. So the hub proteins were identified using connectivity degree analysis.

### Module analysis based on the integrated network

MCODE was used to generate a sub-network for a list of significant network modules. The default threshold was set as following: Degree Cutoff: 2, Node Score Cutoff: 0.2, K-Core: 2, Max. Depth: 100. Subsequently, the GO function and KEGG pathway of significant network modules were enriched.

### Animal model of OA

The male sprague dawley (SD) rats (weight 220 ± 25 g and 8 week-old) purchased from SLAC Laboratory Animal Ltd. Co (Shanghai, China) were used to establish the animal model of OA. All procedures were conformed to the Institutional Animal Care and approved by Committee of Laboratory Animal Center of Nantong University. Rats were randomly divided into the control and model groups (30 rats each group). After acclimatization at the third day, rats in control group were given normal food without any other treatment. The left knee joints of rats in the model group were subjected to anterior cruciate ligament transaction (ACLT) according to the previous study [31], while the right knee joints of rats were served as sham control. Briefly, rats were anesthetized with pentobarbital sodium (30 mg/kg), then shaved and disinfected to expose knee joint. The patella was dislocated laterally and knee joint was buckled as much as possible. After anterior cruciate ligament (ACL) exposed, the ACL was transected with micro-scissors. Next, the joint surface was washed with sterile saline solution, and capsule and skin were sutured. In sham group, the operations were same except for ACLT. Penicillin was injected in a dose of 400,000 units per day for three consecutive days after surgery. Subsequently, rats were sacrificed and their joints were harvested at 8 weeks post-surgery.

### The verification of qRT-PCR

To confirm the above results, expression levels of *QKI*, *MAP 1B* and miR-9 in OA samples (left knee joints) and sham control (right knee joints) were detected using qRT-PCR. Total RNA was extracted from synovial tissues of rats using RNAiso Plus reagent following the manufacturer’s instructions (9109, TAKARA, Japan) under low temperature. Subsequently, the first strand cDNA was prepared from synovial tissues RNA using PrimeScript™RT Master Mix according to the manufacturer’s instructions (RR036A, TAKARA). Subsequently, the rat glyceraldehydes-3-phosphate dehydrogenase (GAPDH) was used as an internal reference. The primers used for *QKI* (Forward primer: AGTACCCCATTGAACCCAGC; Reverse primer: TGTCTGGTAAAACAGTGGGGT) *MAP 1B* (Forward primer: ACGGTAGGGATTACAACG; Reverse primer: GACTCAGGGATGGACTCTT) and GAPDH (Forward primer: AGACAGCCGCATCTTCTTGT; Reverse primer: CTTGCCGTGGGTAGAGTCAT) were designed on rat sequences. The relative amounts of mRNAs were analyzed using Relative Expression Software Tool (REST). In addition, miR-9 (rno-miR-9a-5p-RT: GTCGTATCCAGTGCAGGGTCCGAGGTATTCGCACTGGATACGACTCATAC; JH-rno-miR-9a-5p-Rf: GCGCGCTCTTTGGTTATCTAGCT) was also detected using qRT-PCR. The rat U6 (forward primer: GCTTCGGCAGCACATATACTAAAAT, reverse primer: CGCTTCACGAATTTGCGTGTCAT) was used as an endogenous control. Relative gene expression was assessed by 2^−ΔΔCt^ method.

### Statistical analysis

The gene expression values in the OA and control groups were presented as mean ± standard deviation (X ± SD), and compared using unpaired Student’s t-test by SPSS 22.0 (SPSS Inc., Chicago, IL, USA). *P* < 0.05 was defined to be significantly different.

## Data Availability

The datasets used and analysed during the current study are available from the corresponding author on reasonable request.

## Abbreviations

DEGs: Differentially expressed genes
PPI: Protein-protein interaction
TFs: Transcription factor
GO: Gene Ontology
KEGG: Kyoto Encyclopedia of Genes and Genomes
OA: Osteoarthritis
miRNAs: MicroRNAs
TNF-α: Tumor necrosis factor a
GEO: Gene Expression Omnibus
BP: Biological processes
